# The importance of different frailty domains in a population based sample in England

**DOI:** 10.1186/s12877-019-1411-9

**Published:** 2020-01-15

**Authors:** Solveig A. Arnadottir, Julie Bruce, Ranjit Lall, Emma J. Withers, Martin Underwood, Fiona Shaw, Ray Sheridan, Anower Hossain, Sarah E. Lamb, on behalf of the Pre-FIT Study Group, Sarah E. Lamb, Martin Underwood, Finbarr Martin, Lucy Yardley, Dawn Skelton, Keith Willett, Sandra Eldridge, Anne-Marie Slowther, Sarah Duggan, Julie Bruce, Susie Hennings, Emma Withers, Rhys Mant, Rishpal Rai, Craig Turner, Agata Andrews, Rachael Fearn, Susanne Finnegan, Nicola Walker, Rachel Potter, Ranjit Lall, Claire Hulme, Chris Bojke, Roberta Longo, Susanne Finnegan, Katherine Westacott, Shvaita Ralhan, Ray Sheridan, Jonathan Treml, Ray Sheridan, Jackie Riglin, Harm Gordjin, Ruma Dutta, Jo Burns, Jonathan Treml, Fiona Shaw, John Davison, Ade Willis, Chocks Muthiah, Henry Adjei

**Affiliations:** 1 Department of Physical Therapy, Faculty of Medicine, University of Iceland, Stapi v/Hringbraut, 101, Reykjavik, Iceland; 2 Warwick Clinical Trials Unit, Warwick Medical School, University of Warwick, Coventry, CV4 7AL UK; 3 Older Peoples Medicine, The Newcastle upon Tyne Hospitals NHS Foundation Trust, Freeman Hospital, Freeman Road, High Heaton, Newcastle upon Tyne, NE7 7DN UK; 4 Geriatric Medicine, Royal Devon and Exeter NHS Foundation Trust, Barrack Road, Exeter, Devon EX2 5DW UK; 5 Institute of Statistical Research and Training (ISRT), University of Dhaka, Dhaka, Bangladesh; 6 Nuffield Department of Orthopaedics Rheumatology & Musculoskeletal Sciences, University of Oxford, Windmill Road, Oxford, OX3 7LD UK

**Keywords:** Frailty, Aging, Population characteristics, Sensation, Hearing, Vision

## Abstract

**Background:**

The aim was to estimate the prevalence of frailty and relative contribution of physical/balance, nutritive, cognitive and sensory frailty to important adverse health states (falls, physical activity levels, outdoor mobility, problems in self-care or usual activities, and lack of energy or accomplishment) in an English cohort by age and sex.

**Methods:**

Analysis of baseline data from a cohort of 9803 community-dwelling participants in a clinical trial. The sample was drawn from a random selection of all people aged 70 or more registered with 63 general practices across England. Data were collected by postal questionnaire. Frailty was measured with the Strawbridge questionnaire. We used cross sectional, multivariate logistic regression to estimate the association between frailty domains and known correlates and adjusted for age. Some models were stratified by sex.

**Results:**

Mean age of participants was 78 years (sd 5.7), range 70 to 101 and 47.5% (4653/9803) were men. The prevalence of overall frailty was 20.7% (2005/9671) and there was no difference in prevalence by sex (Odds Ratio 0.98; 95% Confidence Interval 0.89 to 1.08). Sensory frailty was the most common and this was reported by more men (1823/4586) than women (1469/5056; Odds Ratio for sensory frailty 0.62, 95% Confidence Interval 0.57 to 0.68). Men were less likely than women to have physical or nutritive frailty. Physical frailty had the strongest independent associations with adverse health states. However, sensory frailty was independently associated with falls, less frequent walking, problems in self-care and usual activities, lack of energy and accomplishment.

**Conclusions:**

Physical frailty was more strongly associated with adverse health states, but sensory frailty was much more common. The health gain from intervention for sensory frailty in England is likely to be substantial, particularly for older men. Sensory frailty should be explored further as an important target of intervention to improve health outcomes for older people both at clinical and population level.

**Trial registration:**

ISRCTN71002650.

## Background

Frailty is a theoretical state of vulnerability to adverse health outcomes including death, hospitalization and dependence not accounted for by known disease [1]. The core concept is a loss of homeostatic control at a cellular and organ level, and often at a sub- or pre-clinical level [1]. Frailty is described as a multi-dimensional construct, although there is little consensus on the underlying domains [2–4]. Two main frailty models have emerged in last three decades [1, 4], the Fried Phenotypic model of frailty [5], and the Rockwood Cumulative Deficit Model (CDM) [6]. Agreement on a unified model of frailty has been elusive, as have effective population based strategies to minimise frailty.

The Fried model focuses on the role of muscle as the primary reserve organ involved with frailty and hypothesises a direct link between a reduction in muscle mass, strength, metabolic efficiency, related fatigue and exhaustion and slow movement speeds [5]. The CDM is a tally of multiple deficits associated with frailty, including mobility, activities of daily living, sensory and cognitive abilities and presence of some chronic conditions [6].

Regardless of specific definition, different frailty models are unified in seeking to understand pathways that minimize old age disability, prolong active life and delay death [2]. Identification of frail community dwelling older adults may reveal early stages and clinically silent vulnerability to environmental challenges. As frailty can be reversible, it is important for society to identify methods to monitor its prevalence amongst aging adults. Considering frailty based on different domains of body function, may be important in identifying groups who can benefit from diverse types of intervention to enhance and maintain functioning and active participation in society.

As part of a large clinical trial investigating the potential of different population screen and treat strategies for geriatric syndromes (falls, fracture and frailty), we assembled a population based cohort in England in 2011 [7]. We used random sampling of 63 general practices to enrol people aged 70 years and older, requesting that they provide data on their health and functional status by post for a minimum of 18 months and allowed us access to their medical records. We selected the Strawbridge questionnaire to measure frailty [8], as at the time it was the only measure validated for postal data collection. The Strawbridge questionnaire collects data on multiple deficits and groups responses into four domains; this measure is consistent with the CDM concept of frailty.

The aim of this analysis was to estimate the prevalence of frailty, and to explore which domains of frailty were most strongly associated with adverse health outcomes for older adults. The underlying premise was to identify the potential for population based interventions in different domains of frailty.

## Methods

### Study design and participants

Sixty-three general practices from South-West (Devon), Central (Warwickshire/Herefordshire, Cambridge, Worcestershire, Birmingham and Black Country) and Northern England (Newcastle) identified a random sample of community dwelling people aged at least 70 years from their practice lists. People with known terminal illness and life expectancy of less than 6 months were excluded by general practitioners. Between September 2011 and June 2014, potential participants were invited to the clinical trial and completed a baseline postal questionnaire. Practices were asked to provide different practice level fall prevention strategies, but participants were not informed of specific interventions. All practices provided brief postal advice on falls prevention and some practices undertook further screening and intervention (the details are reported elsewhere [7] as we report only baseline data here). The study was approved by the National Research Ethics committee (REC 10/H0401/36). Written informed consent was obtained from all participants.

### Variables

#### Frailty assessment

To assess frailty, we used the 1990’s Strawbridge questionnaire [8] which was based on an early model of frailty, where the underpinning concept was, vulnerability to environmental challenge based upon complex underlying problems. Building on former studies [9–11], the authors combined impairments across four domains of body function into a single outcome and created a multi-dimensional frailty questionnaire. The Strawbridge questionnaire is a relatively simple and user friendly instrument, and although its reliability has not been reported [4], it has established validity for postal administration and self-completion in older community-dwelling adults [4, 8, 12, 13].

The Strawbridge questionnaire [8] includes 16 items that assess frailty across four domains (physical, nutritional, cognitive, and sensory). Four items represent the physical domain (sudden loss of balance, weakness in arms, weakness in legs, dizzy when stand up quickly), two items represent the nutritive domain (loss of appetite, unexplained weight loss) and four items represent the cognitive domain (difficulty paying attention, trouble finding the right word, difficulty remembering things, forgetting where put things). The final six items represent difficulties in the sensory domain (reading newspapers, recognizing a friend across the street, reading signs at night, hearing over the phone, hearing a normal conversation, hearing a conversation in a noisy room). Our mode of administration, scoring and the final Strawbridge frailty classification was according to the original instructions [8]. Thus, for each of the 16 items, participants self-reported if they had experienced problems over the past 12 months and responses were scored: 1 (rarely or never), 2 (sometimes), 3 (often) and 4 (very often). Participants scoring ≥3 (often or very often) on at least one item in any domain were considered to have impairment/frailty within that domain. Participants were classified as frail (overall frailty) if they reported impairments in two or more domains. Missing values were not replaced, and the scoring rules are such that impairment and frailty can be assessed despite some missing values for individual items.

#### Sociodemographic characteristics, health and functioning

We collected self-report data on sex, age, ethnic group, marital status, living arrangements, age leaving full time education (years), height (feet and inches or metres), weight (stones and pounds or kilograms), cognition (clock draw test), self-rated health (SRH), chronic diseases and health conditions (angina/heart trouble; anxiety/depression; cancer; arthritis; chronic lung disease; dementia; diabetes; osteoporosis; Parkinson’s disease; urinary incontinence; stroke (right/left side)). Body Mass Index (BMI kg/m^2^) was calculated using weight and height converted to metric units if appropriate. We used a clock-drawing test [14] to assess global cognition (zero to six point scale whereby higher scores equate to better cognitive ability). SRH was scored on a five point scale (excellent, very good, good, fair, poor) from the 12-Item Short-Form Health Survey (SF-12) [15]. Responses for SRH were collapsed into three categories, 1 = excellent or very good; 2 = good; 3 = fair or poor.

#### Adverse health states

We asked seven questions about adverse health states. Falls were defined using an internationally agreed definition, by recall over the previous 12 months [16]. Participants were asked “are you able to get out and about on foot outside the house” and “on average how many hours a day do you spend walking?” We classified people as having poor outdoor mobility if they were unable to get out and about on foot outside the house unaided. Those walking less than 1 h per day were considered to have low physical activity. We used the three-level version of the EuroQol five-dimensional questionnaire (EQ-5D-3L) [17] to identify participants with limitation in self-care (i.e. some problem or inability to wash or dress themselves), and restricted participation was defined as having some problems or being unable to perform usual activities.

Finally, we used the following two questions from the Short Form 12 Health Survey (SF-12) instrument [15]. We asked, “how much of the time during the past four weeks did you have a lot of energy?” and classed those with no or little energy as having low energy. We asked, “during the past four weeks, have you accomplished less than you would like as a result of your physical health?” and used responses to identify participants who accomplished less than they liked, all or most of the time.

### Statistical analysis

We present descriptive statistics using means, standard deviations (sd) and/or frequency distributions and proportions. Prevalence of frailty by each domain and overall frailty were compared by sex using odds ratio (OR) and 95% confidence intervals (95% CI), with and without adjustment for age. We analysed frailty prevalence by sex (Pearson’s chi-square test) in the following age groups: 70–74, 75–79, 80–84, 85–89, 90+ years. We used multivariate logistic regression to estimate the independent contribution of different domains of frailty to adverse health states with adjustment for age. We selected factors for inclusion in multivariate models using univariate analysis and a *P* value of < 0.1. Statistical significance in the final models was set at *P* < 0.05. Stata/SE (version 15.1) was used for all statistical analyses.

## Results

### Participant characteristics

A total of 29,010 people was invited to take part in the clinical trial, of these 9803 provided valid data and consent (response rate = 33.8%; 9803/29010).

Table 1 presents descriptive characteristics of responding participants. Age ranged from 70 to 101 years and 47.5% (4653/9803) were men. On average, men were slightly younger, more likely to be married or cohabiting, less likely to be living alone and were less likely to report adverse health outcomes than women.

**Table 1:** Characteristics of participants

Characteristics	n^a^	Total (*N* = 9803)	Men (*n* = 4653)	Women (*n* = 5150)	*P* value^c^
Age, M ± sd (min-max)	9803	77.9 ± 5.7 (70.0–101.0)	77.7 ± 5.6 (70.0–100.7)	78.1 ± 5.8 (70.0–101.0)	< 0.001
Age category, n (%):	9803				0.003
70–74		3670 (37.5)	1795 (38.6)	1875 (36.4)	
75–79		2885 (29.4)	1376 (29.6)	1509 (29.3)	
80–84		1951 (19.9)	909 (19.5)	1042 (20.2)	
85–89		983 (10.0)	441 (9.5)	542 (10.5)	
90+		314 (3.2)	132 (2.8)	182 (3.6)	
Ethnic group, white	9725	9630 (99.0)	4565 (98.6)	5065 (99.4)	< 0.001
Married or cohabiting, n (%)	9765	6170 (63.2)	3638 (78.5)	2532 (49.3)	< 0.001
Living alone, n (%)	9745	3217 (33.0)	902 (19.5)	2315 (45.2)	< 0.001
Age when left full time education, M ± sd (min-max)	9648	16.8 ± 4.7 (10.0–79.0)	16.9 ± 4.7 (10.0–79.0)	16.7 ± 4.6 (10.0–78.0)	0.020
Body Mass Index (BMI), kg/m^2^, M ± sd (min-max)	9480	26.5 ± 4.6 (11.6–57.5)	26.6 ± 4.2 (12.8–49.6)	26.3 ± 5.0 (11.6–57.5)	0.006
Clock-drawing,^b^ M ± sd (min-max)	9621	5.5 ± 0.9 (0–6)	5.6 ± 0.9 (0–6)	5.5 ± 1.0 (0–6)	0.009
Clock-drawing, score of 6, n (%)	9621	6865 (71.4)	3279 (71.8)	3586 (71.0)	0.144
Self-rated health, n (%)	9718				< 0.001
Excellent or very good		4314 (44.4)	2175 (47.1)	2139 (41.9)	
Good		3470 (35.7)	1570 (34.0)	1900 (37.2)	
Fair or poor		1934 (19.9)	870 (18.9)	1064 (20.9)	
Health conditions, n (%):					
Angina or heart troubles	9342	2697 (28.9)	1493 (33.2)	1204 (24.9)	< 0.001
Anxiety, depression, other	9116	1114 (12.2)	412 (9.4)	702 (14.8)	< 0.001
Arthritis (RA or OA)	9405	4403 (46.8)	1643 (37.0)	2760 (55.7)	< 0.001
Cancer (active)	9042	1316 (14.6)	696 (15.9)	620 (13.3)	< 0.001
Chronic lung disease	9030	641 (7.1)	344 (7.9)	297 (6.4)	0.005
Dementia	8885	68 (0.8)	46 (1.1)	22 (0.5)	0.001
Diabetes	9135	1403 (15.4)	770 (17.5)	633 (13.4)	< 0.001
Osteoporosis	9012	1172 (13.0)	195 (4.6)	977 (20.7)	< 0.001
Parkinson’s disease	8867	93 (1.1)	58 (1.3)	35 (0.8)	0.006
Urinary incontinence	8989	933 (10.4)	396 (9.2)	537 (11.5)	< 0.001
Stroke, with affected side	452	174 (38.5)	95 (39.9)	79 (36.9)	0.513
Adverse health states, n (%):					
At least one fall in past year	9737	3150 (32.4)	1382 (29.9)	1768 (34.6)	< 0.001
No walking or less than 1 h/day spent walking	9754	2566 (26.3)	1227 (26.5)	1339 (26.2)	0.733
Unable to get unaided, out and about on foot outside the house	9763	1952 (20.0)	702 (15.1)	1250 (24.4)	< 0.001
Some problems or unable to wash or dress oneself	9710	931 (9.6)	418 (9.1)	513 (10.1)	0.094
Some problems or unable to perform usual activities	9708	3108 (32.0)	1295 (28.1)	1813 (35.6)	< 0.001
Does not have a lot of energy, all or most of the time	9685	5038 (52.0)	2152 (46.7)	2886 (56.8)	< 0.001
Accomplishes less than likes, all or most of the time, due to physical health	9682	1375 (14.2)	806 (15.9)	569 (12.4)	< 0.001

^a^Number of participants (n), differs between rows due to missing values; ^b^ Clock-drawing test [14] results indicate a global cognitive function. Scores can range from 0 to 6 and higher score indicates better cognitive function; ^c^ Continuous variables: *P* value for t-test (Age, Age when left full time education, BMI, Clock-drawing); Ordinal variables: *P* value for Mann-Whitney U test (Age category, Self-rated health); Binary variables: *P* value for chi-square test (all other variables)

### Prevalence of frailty

**Fig. 1:**
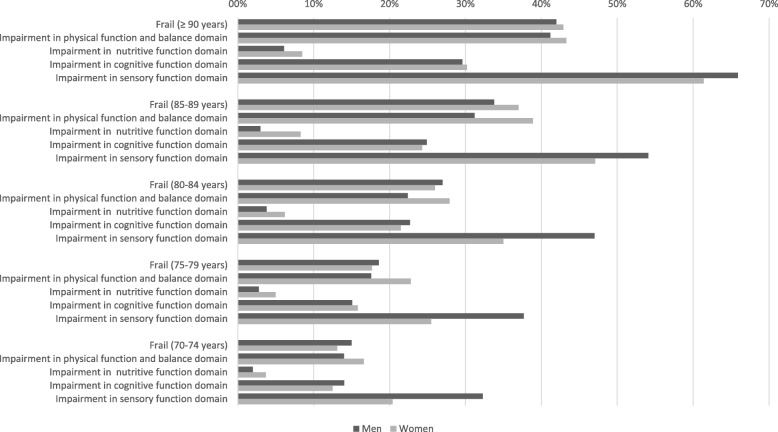
Prevalence of frailty and underlying impairments in four domains, by age group and sex. Strawbridge frailty definition is based on impairment in two or more of the four underlying physical, nutritive, cognitive and sensory domains. An impairment is documented if a participant reports that he/she, over the past 12 months, has often or very often experienced a problem in that domain

Based on observed values, frailty status was identified for 9671/9803 (98.7%) of the participants. Due to some missing values across individual items, frailty status was missing for 1.3% of participants. Completion rate by each frailty domain varied, hence the denominator varies. Figure 1 demonstrates frailty across successive age bands by sex. Sensory frailty was more prevalent in men than women across all age bands, although this margin narrowed in those over 90 years of age.

The prevalence of overall frailty was 20.7% (2005/9671), and there was no difference in overall frailty between men and women (960/4592, 20.9% versus 1045/5079, 20.6%; respectively). The unadjusted odds ratio (OR) for overall frailty, with men as reference, was 0.98 (95% CI 0.89–1.08) and this estimate did not change when age-adjusted. Sensory frailty was more prevalent in men (1823/4587, 39.7%) than women (1469/5056, 29.1%) whilst physical frailty was more prevalent in women (1223/5098, 24%) compared to men (881/4607, 19.1%). Nutritive frailty was almost double in women (266/5101, 5.2%) compared to men (129/4599, 2.8%). Cognitive frailty was similar between men (808/4612, 17.5%) and women (876/5110, 17.1%). Table 2 presents more detailed prevalences and odds of frailty by sex.

**Table 2:** Prevalence and odds of frailty, impairments in frailty domains by Strawbridge item

Variables based on the Strawbridge questionnaire	Prevalence of frailty and impairment by domain N (%)			Odds Ratio^a^ (95% Confidence Intervals) (comparing women to men, men as reference)	
	All participants *N* = 9803	Men *n* = 4653	Women *n* = 5150	Crude	Adjusted for age
Frail^b^ (data available for *n* = 9671)	2005 (20.7%)	960 (20.9%)	1045 (20.6%)	0.98 (0.89–1.08)	0.94 (0.85–1.04)
Impairment in physical frailty domain^c^ (*n* = 9705)	2104 (21.7%)	881 (19.1%)	1223 (24.0%)	1.33 (1.21–1.47)*	1.30 (1.18–1.44)*
Sudden loss of balance^d^ (*n* = 9739)	643 (6.6%)	244 (5.3%)	399 (7.8%)	1.52 (1.29–1.79)*	1.46 (1.24–1.73)*
Weakness in arms (*n* = 9721)	799 (8.2%)	297 (6.4%)	502 (9.8%)	1.58 (1.36–1.84)*	1.55 (1.34–1.80)*
Weakness in legs (*n* = 9729)	1457 (15.0%)	630 (13.7%)	827 (16.2%)	1.22 (1.09–1.36)*	1.19 (1.06–1.33)*
Dizziness when standing up quickly (*n* = 9735)	614 (6.3%)	275 (6.0%)	339 (6.6%)	1.122 (0.952–1.322)	1.09 (0.92–1.29)
Impairment in nutritive frailty domain (*n* = 9700)	395 (4.1%)	129 (2.8%)	266 (5.2%)	1.90 (1.54–2.36)*	1.86 (1.50–2.31)*
Loss of appetite (*n* = 9740)	354 (3.6%)	112 (2.4%)	242 (4.7%)	1.99 (1.59–2.50)*	1.95 (1.55–2.45)*
Unexplained weight loss (*n* = 9707)	90 (0.9%)	31 (0.7%)	59 (1.2%)	1.73 (1.12–2.67)*	1.68 (1.08–2.60)*
Impairment in cognitive frailty domain (*n* = 9722)	1684 (17.3%)	808 (17.5%)	876 (17.1%)	0.97 (0.88–1.08)	0.95 (0.85–1.06)
Difficulty paying attention (*n* = 9732)	220 (2.3%)	128 (2.8%)	92 (1.8%)	0.64 (0.49–0.84)*	0.63 (0.48–0.83)*
Trouble finding the right word (*n* = 9741)	773 (7.9%)	333 (7.2%)	440 (8.6%)	1.21 (1.04–1.40)*	1.18 (1.02–1.37)*
Difficulty remembering things (*n* = 9747)	1045 (10.7%)	539 (11.7%)	506 (9.9%)	0.83 (0.73–0.94)*	0.80 (0.71–0.92)*
Forgetting where put something (*n* = 9750)	1104 (11.3%)	524 (11.3%)	580 (11.3%)	1.00 (0.88–1.13)	0.97 (0.86–1.10)
Impairment in sensory frailty domain (*n* = 9643)	3292 (34.1%)	1823 (39.7%)	1469 (29.1%)	0.62 (0.57–0.68)*	0.59 (0.54–0.64)*
Difficulty reading a newspaper (*n* = 9735)	337 (3.5%)	165 (3.6%)	172 (3.4%)	0.94 (0.76–1.17)	0.87 (0.70–1.09)
Difficulty recognizing friend across street (*n* = 9715)	261 (2.7%)	120 (2.6%)	141 (2.8%)	1.06 (0.83–1.36)	0.98 (0.77–1.26)
Difficulty reading signs at night (*n* = 9627)	550 (5.7%)	216 (4.7%)	334 (6.6%)	1.44 (1.21–1.72)*	1.39 (1.16–1.66)*
Difficulty hearing over the phone (*n* = 9727)	1291 (13.3%)	729 (15.8%)	562 (11.0%)	0.66 (0.58–0.74)*	0.63 (0.56–0.71)*
Difficulty hearing a normal conversation (*n* = 9732)	1080 (11.1%)	615 (13.3%)	465 (9.1%)	0.65 (0.57–0.74)*	0.63 (0.55–0.71)*
Difficulty hearing conversation in a noisy room (*n* = 9730)	2919 (30.0%)	1683 (36.5%)	1236 (24.2%)	0.56 (0.51–0.61)*	0.53 (0.48–0.58)*

Number of participants (n), differs between rows due to missing values; ^a^ Odds of frailty, impairment or difficulty, using males as the reference; ^b^ Overall frailty is defined as having impairment in ≥2 out of 4 frailty domains; physical, nutritive, cognitive, sensory; ^c^ For the four domains (physical, nutritive, cognitive and sensory), impairment is defined as having difficulties in at least one item within the domain; ^c^ For each of the 16 items on the Strawbridge questionnaire, participants are asked if they have experienced difficulties over the past 12 months. For the calculation of frailty score, having difficulties on an item is defined as a rating of “often” or “very often” (≥3); * Indicates a significant difference in frailty scores based on sex

### Association with adverse health states

In all participants, the overall frailty was strongly associated with having fallen in the last year, poor outdoor mobility, lower physical activity level, problems with self-care, restricted participation in usual activities, having less energy and lower accomplishment. For both sexes, this association was independent of age and varied slightly depending on the adverse health state (Table 3).

**Table 3:** Frailty and impairments by frailty domain, associated with seven adverse health states

	All	Men	Women
	Adjusted Odds Ratio (95% CI)	Adjusted Odds Ratio (95% CI)	Adjusted Odds Ratio (95% CI)
At least one fall in past year			
Frailty^a^	2.83 (2.55–3.14)	3.10 (2.66–3.60)	2.66 (2.31–3.07)
Frailty domain:^b^			
Physical impairment	2.56 (2.30–2.87)	3.05 (2.57–3.60)	2.21 (1.91–2.56)
Nutritive impairment	1.84 (1.47–2.29)	1.64 (1.11–2.42)	1.93 (1.47–2.55)
Cognitive impairment	1.62 (1.44–1.83)	1.72 (1.43–2.05)	1.53 (1.30–1.81)
Sensory impairment	1.19 (1.08–1.31)	1.22 (1.05–1.41)	1.22 (1.06–1.40)
Unable to get unaided, out and about on foot outside the house			
Frailty^a^	4.18 (3.72–4.69)	4.96 (4.15–5.94)	3.90 (3.34–4.57)
Frailty domain:			
Physical impairment	9.11 (7.99–10.38)	10.55 (8.57–13.00)	8.06 (6.80–9.55)
Nutritive impairment	2.29 (1.80–2.91)	1.85 (1.21–2.84)	2.43 (1.77–3.33)
Cognitive impairment	0.92 (0.79–1.07)	0.97 (0.76–1.23)	0.88 (0.72–1.09)
Sensory impairment	1.12 (0.99–1.28)	1.18 (0.96–1.45)	1.23 (1.04–1.47)
No walking or less than 1 h/day spent walking			
Frailty^a^	2.46 (2.21–2.73)	2.54 (2.18–2.96)	2.38 (2.05–2.76)
Frailty domain:^b^			
Physical impairment	2.32 (2.07–2.60)	2.42 (2.04–2.87)	2.28 (1.96–2.65)
Nutritive impairment	2.16 (1.73–2.70)	2.08 (1.42–3.05)	2.25 (1.71–2.96)
Cognitive impairment	1.24 (1.09–1.41)	1.26 (1.05–1.52)	1.22 (1.02–1.46)
Sensory impairment	1.30 (1.17–1.45)	1.26 (1.08–1.46)	1.31 (1.13–1.53)
Some problems or unable to wash or dress oneself			
Frailty^a^	7.26 (6.26–8.41)	8.40 (6.73–10.48)	6.47 (5.30–7.89)
Frailty domain:^b^			
Physical impairment	8.52 (7.22–10.05)	9.70 (7.57–12.44)	7.61 (6.10–9.50)
Nutritive impairment	2.25 (1.75–2.89)	1.99 (1.30–3.06)	2.43 (1.78–3.31)
Cognitive impairment	1.90 (1.61–2.26)	2.35 (1.83–3.01)	1.58 (1.25–2.00)
Sensory impairment	1.31 (1.11–1.54)	1.27 (0.99–1.64)	1.32 (1.05–1.65)
Some problems or unable to perform usual activities			
Frailty^a^	5.90 (5.28–6.58)	6.34 (5.42–7.43)	5.72 (4.91–6.68)
Frailty domain:^b^			
Physical impairment	9.43 (8.34–10.66)	9.37 (7.79–11.29)	9.21 (7.81–10.87)
Nutritive impairment	2.61 (1.99–3.42)	2.56 (1.61–4.08)	2.43 (1.74–3.40)
Cognitive impairment	1.51 (1.32–1.73)	1.51 (1.24–1.83)	1.50 (1.25–1.81)
Sensory impairment	1.62 (1.46–1.81)	1.71 (1.46–2.01)	1.72 (1.47–2.01)
Does not have a lot of energy, all or most of the time			
Frailty^a^	5.86 (5.15–6.67)	6.14 (5.14–7.32)	5.91 (4.88–7.17)
Frailty domain:^b^			
Physical impairment	6.24 (5.41–7.20)	5.82 (4.73–7.16)	6.36 (5.21–7.77)
Nutritive impairment	5.06 (3.43–7.46)	6.98 (3.41–14.28)	3.98 (2.45–6.19)
Cognitive impairment	2.27 (1.99–2.63)	2.43 (2.00–2.95)	2.14 (1.75–2.62)
Sensory impairment	1.60 (1.41–1.72)	1.51 (1.31–1.74)	1.91 (1.64–2.23)
Accomplishes less than likes, all or most of the time, due to physical health			
Frailty^a^	6.74 (5.94–7.65)	7.64 (6.30–9.27)	6.25 (5.28–7.39)
Frailty domain:^b^			
Physical impairment	8.27 (7.20–9.49)	8.74 (7.05–10.83)	7.76 (6.47–9.30)
Nutritive impairment	3.26 (2.54–4.18)	3.64 (2.37–5.58)	3.96 (2.18–4.02)
Cognitive impairment	1.52 (1.30–1.78)	1.60 (1.26–2.02)	1.46 (1.18–1.81)
Sensory impairment	1.40 (1.22–1.61)	1.40 (1.12–1.74)	1.47 (1.22–1.78)

^a^Frailty definition is based on impairment in ≥2 out of the 4 underlying physical, nutritive, cognitive and sensory frailty domain (0 = not frail; 1 = frail and 0 = no impairment; 1 = impairment); ^b^ Each impairment adjusted for the others; All models are based on multivariable logistic regression, adjusting for the effects of age (continuous variable)

Table 3 also presents the association between each frailty domain and each adverse health state. Overall, physical frailty was strongly associated with all adverse health states. The odds ratio varied across health states, from OR 9.43 (95% CI 8.34–10.66) for problems with restricted participation in usual activities to OR 2.32 (95% CI 2.07–2.60) for decreased physical activity level. Nutritive frailty was also significantly associated with all adverse health states and the association varied from OR 5.06 (95% CI 3.43–7.46) for lower energy to OR 1.84 (95% CI 1.47–2.29) for falls. Cognitive and sensory frailty demonstrated smaller but consistent associations across the adverse health states. The only health state not associated with cognitive frailty was outdoor mobility. Sensory frailty was associated with all states, although the association with poor outdoor mobility and limitation in self-care was only statistically significant amongst women. Other patterns of association were comparable in both women and men.

## Discussion

In this analysis of baseline data from a cohort of older adults recruited to a falls prevention study, we found that the overall prevalence of frailty was 20.7% using the Strawbridge questionnaire, with no difference between men and women. Sensory impairments were the most common, particularly common in men, and had modest but important associations with most adverse health states. Physical and nutritive frailty were less prevalent, but more common in women, and were also strongly associated with adverse health states. The findings demonstrate that impairments contributing to frailty are a substantial problem in community dwelling older people in England.

Frailty prevalence amongst older community-dwelling adults has been shown to vary depending on definition, age and health of study cohorts [18]. The underlying definition influences the choice of specific items included in frailty assessments, and hence frailty prevalence. In one large French cohort using the Strawbridge questionnaire in people aged 58 to 73 years [12], the authors demonstrated how inclusion of a sensory domain in a frailty screening instrument increases frailty prevalence, compared to when the sensory domain was excluded. The Strawbridge questionnaire is weighted towards sensory impairment, with the inclusion of six items on vision and hearing (compared to four physical, four cognitive and two nutritive). This allows exploration of the potential contribution of sensory deficits to health and functioning [19]. One sensory question asks about difficulty in understanding speech in challenging environments, such as a noisy room. This ‘listening-in-noise’ difficulty has been identified in younger people with normal or near-normal hearing thresholds [20] and may be an early predictor of future deficits. This allows for a broader assessment of frailty [12], which again may assist in capturing and assisting people well before they enter potential adverse health states.

In comparison to other studies that have used the Strawbridge questionnaire, our estimate of overall frailty prevalence (20.7%) was almost identical to the US Health and Retirement Study (20.3%) based on a population sample in 2004, aged 65 years and older, without stroke, depression, or moderate to severe cognitive impairments [2]. In the original publication by Strawbridge et al. [8], frailty prevalence in Alameda County US in 1994 was 26.1%, which may reflect inclusion of participants who had moved into an institution. Moreover, the French GAZEL cohort study more recently reported 18.6% frailty, in a 70–73 years old group, using the Strawbridge questionnaire [12]. Our estimates of impairment rate within each frailty domain are comparable with previous studies [8, 12] and the association between frailty and age is well known from other cross-sectional and longitudinal studies [2, 18, 21].

Our results differ from majority of research which reveal higher prevalence of frailty among women compared to men, but these studies tend to use questionnaires that emphasise the physical and nutritive components of frailty [18, 22–26] or chronic conditions recorded in medical records [27]. The inclusion and number of sensory items makes the Strawbridge questionnaire more sensitive to impairments which are more common in men than women [8, 28].

In addition to the prevalence data, our analysis supports the hypothesis that each of the frailty domains are an important construct, as the adverse health states we examined are consistently identified as a high priority by older people [29]. The relationship between physical and nutritive frailty and adverse health states is expected, based on other observations [5]. The relationship between sensory frailty and the adverse health states is consistent with sensory deficits as potential contributors to vulnerability for developing increased dependency [30]. Moreover, Strawbridge’s sensory frailty has been shown to predict hospitalisation and, in the same study, both sensory and cognitive frailty were predictive of future disability [12]. Our findings are based on cross-sectional data thus a causal pathway between frailty and adverse states cannot be implied from this single time point.

Our results do indicate a potential for future research on population based interventions in different domains of frailty. The Strawbridge questionnaire [8] focuses on limb weakness and balance problems in the physical frailty domain, and exercise interventions can be effective in improving strength and balance [31]. The sensory domain reflects impairments of vision and hearing, and these can be challenging to ameliorate. Yet, provision of services and effective interventions aimed at people with visual and hearing impairments (such as cataract surgery and hearing aids) have the potential to improve social participation and quality of life across large number of older people [32–34]. Therefore, improving treatments for age-associated visual and hearing loss should be a public health priority.

The strength of our work is that the data is drawn from a large population random sample recruited to a clinical trial. A range of GP practices from rural and urban England contributed but a limitation is that these were not a random selection of all English practices. However, they were representative of the anticipated mix of practices in terms of socioeconomic and demographic mix. About one third of older people approached to participate in the study agreed (uptake 34%). Whilst there were no major differences in age and sex between people agreeing to participate and those not, we have limited data to assess selection bias. The sample was almost identical to the expected age and sex for England [35]. The proportion of people referring to themselves as white was 99%, being higher than estimates from the 2011 census which estimates 98% at age 90 years and 95% at age 70 years [35].

The study cohort was assembled for two purposes including future epidemiological research and a cluster randomised controlled trial of screen and treat strategies for geriatric syndromes implemented at the general practice levels. Cohort randomised controlled trials [36] are becoming increasingly common and use a range of different designs which first assemble a cohort and then invite some participants to test interventions using those who are not invited as controls. We used a cluster trial design to assign interventions, with randomisation and intervention at the practice level. Participants were not aware of the interventions being tested by their practice. Baseline data was collected from all participants prior to practice allocation, and hence should not have affected any of the associations or estimates reported in this paper.

While data return and completion were excellent, we may have underestimated the prevalence of sensory and cognitive impairment as these are likely to be associated with questionnaire completion. We asked that participants completed the postal questionnaire themselves, and we accept that it is possible that carers or associates may have completed the questionnaire. This seems unlikely given the pattern of responses we observed and the qualitative narrative that participants provided alongside their questionnaire responses.

Finally, a continuous frailty scale might be more sensitive, but measures which capture the frequency of problems over a longer time period are more predictive than isolated measures of performance [37]. A disadvantage of the Strawbridge assessment of frailty is that it is not widely used, but it remains one of the few instruments that can be collected by postal questionnaire [4, 38] and it is one of the instruments without items on comorbidity or disability [4]. Although there are numerous frailty scales currently in use, research is needed on their reliability, validity and usefulness in both community and clinical settings [4, 38].

## Conclusions

Frailty is a prevalent condition in the population of English people. Sensory frailty is the most common form of frailty and affects more men than women. Although sensory frailty has a more modest association with adverse health states than physical frailty, the potential benefits of clinical and population based intervention to ameliorate sensory frailty should not be overlooked.

## Data Availability

The data that support the findings of this study are available from the Chief Investigator Professor Sarah E Lamb (sarah.lamb@ndorms.ox.ac.uk) upon reasonable requests for data sharing.

## Abbreviations

BMI: Body Mass Index
CDM: Cumulative Deficit Model
CI: Confidence interval
EQ-5D-3L questionnaire: Three-level version of the EuroQol five-dimensional questionnaire
OR: Odds ratio
SD: Standard deviation
SF-12: 12-Item Short-Form Health Survey
SRH: Self Rated Health
